# Impact of Lifestyle Intervention on Long-Term Beverage Intake in Children with Overweight and Obesity: A 3-Year Follow-Up Study

**DOI:** 10.3390/nu18010147

**Published:** 2026-01-01

**Authors:** Martin Emil Jørgensen, Dorthe Dalstrup Pauls, Daniel Borch Ibsen, Jens Meldgaard Bruun

**Affiliations:** 1 Steno Diabetes Center Aarhus, Aarhus University Hospital, 8200 Aarhus, Denmark; majo@biomed.au.dk (M.E.J.); dbi@ph.au.dk (D.B.I.); jens.bruun@clin.au.dk (J.M.B.); 2 Department of Clinical Medicine, Aarhus University, 8000 Aarhus, Denmark; 3 Danish National Center for Obesity, 8200 Aarhus, Denmark; 4 Department of Public Health, Aarhus University, 8000 Aarhus, Denmark

**Keywords:** lifestyle intervention, childhood obesity, overweight and obesity, dietary intake, children and adolescents, sugar-sweetened beverages, non-caloric beverages, milk, water, chocolate milk

## Abstract

**Background**: Higher intake of sugar-sweetened beverages (SSBs) increases the risk of childhood obesity, while the effects of non-caloric beverages (NCBs) and dairy beverages remain inconsistent. This study investigated changes in beverage intake following a 10-week lifestyle camp and explored associations between changes in beverage intake and anthropometric measures. **Methods**: Children from two camp sites and aged 7–14 years (n:190) with overweight/obesity were included and pooled for the present study. Beverage intake was assessed by a Food Frequency Questionnaire at baseline, at 10 weeks, and at a one- and three-year follow-up. Anthropometry was assessed at the same timepoints. **Results**: Compared to baseline, participants had lower odds of reporting a high intake of SSBs (OR: 0.14, 95%-CI: 0.07; 0.28), NCBs (OR: 0.19, 95%-CI: 0.11; 0.34) and chocolate milk (OR: 0.18, 95%-CI: 0.09; 0.36) at 10 weeks, relative to a low intake, and were more likely to report a high intake of water. One and three years after the camp, the changes attenuated, reaching baseline levels for water, SSBs, and NCBs at the three-year follow-up. Across time, only weak to moderate correlations were observed between changes in beverage intake and BMI-SDS, body fat (%), and skeletal muscle mass (Spearman’s rho = +/− 0.19–0.31). **Conclusions**: The lifestyle camp intervention effectively improved beverage intake among children with overweight/obesity; however, the changes were not sustained, emphasizing the need for long-term support to maintain the health benefits. Without a control group, it is not possible to determine whether these changes reflect natural variations in beverage intake.

## 1. Introduction

The global prevalence of overweight and obesity is steadily rising, posing major global health challenges [1]. According to data from the World Health Organization (WHO), 390 million children and adolescents (aged 5–19 years) were living with overweight in 2022, and of those, 160 million were living with obesity [2]. Childhood obesity is strongly associated with obesity in adulthood [3], which significantly increases the risk of a wide range of non-communicable diseases, including type 2 diabetes, cardiovascular diseases, and up to 15 obesity-specific cancers [4]. Moreover, overweight and obesity in childhood and adolescence increases the likelihood of depression and low self-esteem, with negative impact on emotional well-being, thereby reducing the likelihood of achieving good physical and psychological health, which may impair social functioning, including a reduced tendency to form peer relationships [5,6].

Dietary intake, including beverage consumption, plays a significant role in the development of childhood obesity [7,8]. In most countries, children and adolescents are recommended to reduce the intake of sugar-sweetened beverages (SSBs) and to consume water and milk on a daily basis to secure growth while developing healthy dietary habits [9]. Current evidence consistently shows that a higher intake of water is associated with favorable anthropometric features in children, such as lower Body Mass Index (BMI) and body fat [10,11]. On the contrary, a higher intake of SSBs is associated with less favorable anthropometric features in children, such as higher BMI, body fat, and waist circumference [12,13,14,15,16], while the association with non-caloric beverages (NCB) and milk yield contradicting results [8,17,18,19,20,21].

Few studies have examined the long-term effects of interventions aimed at reducing unhealthy beverage consumption in children. A cluster randomized controlled trial (RCT) in 12-year-olds demonstrated a reduction in the intake of SSBs following a 6-month school-based intervention compared to controls, and a further decrease in SSBs was observed from post-intervention to the 19-month follow-up [22]. Similarly, an RCT involving 15-year-olds reported a reduction in SSB intake after a 1-year multicomponent intervention followed by an increase at the 2-year follow-up, although intake of SSBs remained slightly lower in the intervention group compared to the control group [23]. Another RCT reported that 14-year-old boys who participated in an 4- to 12-week text-message-based intervention exhibited a trend toward reduced SSB intake at a 6-month follow-up (*p* = 0.08), whereas no effect was observed in girls (*p* = 0.50), although the effect did not persist at the 18-month follow-up [24]. Collectively, available evidence indicates that both school- and home-based interventions can promote reductions in SSB intake among children and adolescents, and in some cases, these effects persist after the intervention. However, findings on long-term effects are sparse and inconsistent, and studies with follow-up periods beyond two years are needed.

The aim of this study is to investigate short- and long-term changes in beverage intake (i.e., water, SSBs, NCBs, dairy milk, and chocolate milk) in children with overweight and obesity attending a 10-week multicomponent lifestyle camp. Additionally, the study aims to investigate whether changes in beverage intake are associated with changes in anthropometric measures across time.

## 2. Materials and Methods

The data utilized in this study were derived from the foundational work of the “Childhood Obesity-Prevention of Diabetes Through Changed Eating Patterns Study” referred to as the COPE study [25]. The COPE study is a prospective, non-randomized, controlled study designed to investigate the effects of a higher protein diet and to evaluate the overall effect of a 10-week multicomponent lifestyle camp on child health and well-being. The COPE study included two camp sites. One camp was assigned as the intervention group (higher protein diet) and one as the control group (standard protein diet in accordance with national recommendations) [26]. No differences were observed between the intervention and control group in BMI standard deviation score (BMI-SDS) during the intervention (*p* = 0.24). In addition, reported beverage intake did not differ across time between the groups, supporting their pooling for the present study [25].

The complete study design has been described in detail elsewhere [25]. Briefly, the lifestyle camps offer a structured 10-week program designed to support children aged 7 to 14 years who are experiencing adverse life circumstances such as loneliness, bullying, and/or living with and struggling with overweight or obesity. The camps aim to improve health and well-being and encourage healthier lifestyle habits through a range of structured activities and support initiatives. Participation is free of charge for the families. To attend camp, caregivers are required to complete an application form detailing the child’s physical and psychological challenges, as well as their motivation for attending. In addition, the child’s general practitioner must submit a corresponding medical form that, in addition to confirming the caregiver’s application, includes relevant health information and any potential diagnoses.

Children were excluded from the present study if they failed to complete a Food Frequency Questionnaire (FFQ) at baseline and/or did not provide anthropometric data at baseline. Children with a chronic illness requiring a special diet and children with normal weight at baseline (BMI-SDS ≤ 1 SD) were also excluded.

### 2.1. Questionnaires

Participants completed several questionaries with assistance from their parents at baseline, after 10 weeks (end of camp), and at a one-year and three-year follow-up.

Dietary intake was assessed with ‘The Children’s Eating Habits Questionnaire-FFQ’, which was intended as a screening tool for assessing dietary intake related to childhood overweight, obesity, and general health [27]. As part of The COPE study, the FFQ was translated, and a few food items were added for cultural adaptation. The final version of the FFQ included a variety of food and beverage items, with a total of 33 different items. The questions were formulated to capture participants’ behavior during a typical week within the past month. Each dietary product was assessed using a 9-point-Likert-scale, with options ranging from “never/less than once per week” to “four or more times per day”. In the present study, only beverage items were assessed, including water, SSBs (juice, soft drink, fruit drink concentrate), NCBs (no sugar/artificial sugared beverages), dairy milk, and chocolate milk.

### 2.2. Anthropometry

Body weight (kg), body fat (%), and skeletal muscle mass (kg) were assessed using bioelectrical impedance analysis (InBody Model 270). In addition, height was measured using a fixed wall measuring tape. BMI-SDS was calculated using the World Health Organization AnthroPlus software for Windows, version 1.0.4. For children aged 5–19 years, a BMI-SDS > 1 SD is categorized as overweight and a BMI > 2 SD is categorized as obesity [28].

All assessments were conducted by camp staff at baseline and 10 weeks (end of camp) and at a one-year and three-year follow-up assessment.

### 2.3. Statistical Analysis

The data were analysed using STATA MP version 18.5 with a significance threshold set at *p* < 0.05. Continuous data are presented as mean ± SD, and categorical data as absolute numbers and percentages (%).

The distribution of responses for beverage intake was skewed, with some categories receiving few or no responses. Consequently, beverage intake was divided into two categories: ‘low intake’ and ‘high intake’, and ‘don’t know’ responses were excluded from all analyses. The categories were constructed as presented in Table 1.

The categorization of low vs. high beverage intake was based upon Danish national dietary guidelines on water, dairy milk, SSBs, and NCBs for children [26]. For SSBs and NCBs, intake was approximated based on recommendations for children aged 7 to 10 years (33 cL per week) and 11 to 17 years (50 cL per week), as the questionnaire did not specify the exact volume of beverage consumed. Due to the absence of specific recommendations for chocolate milk, as well as the skewed distribution of answers, chocolate milk was classified pragmatically with ‘low intake’ corresponding to ‘no intake’ and ‘high intake’ corresponding to ‘some intake’, being at least once a week. The beverage intake categories formed the basis for all analyses.

Associations between intake categories and anthropometric data at baseline were assessed using Spearman’s rank correlation tests, presenting correlation coefficients (rₛ) and *p*-values. To investigate changes across time in the probability of reporting a high versus low beverage intake, logistic regression analyses were performed with results presented as odds ratios (ORs) and 95% confidence intervals (95% CIs), using low intake as the reference category in all analyses. Furthermore, to explore whether changes in beverage intake was associated with changes in anthropometric data across time, Spearman’s rank correlation tests were used with results presented as correlation coefficients (rₛ) and *p*-values. Analyses included all participants with at least one valid assessment over the three years, using all available data without imputation.

## 3. Results

**Figure 1:**
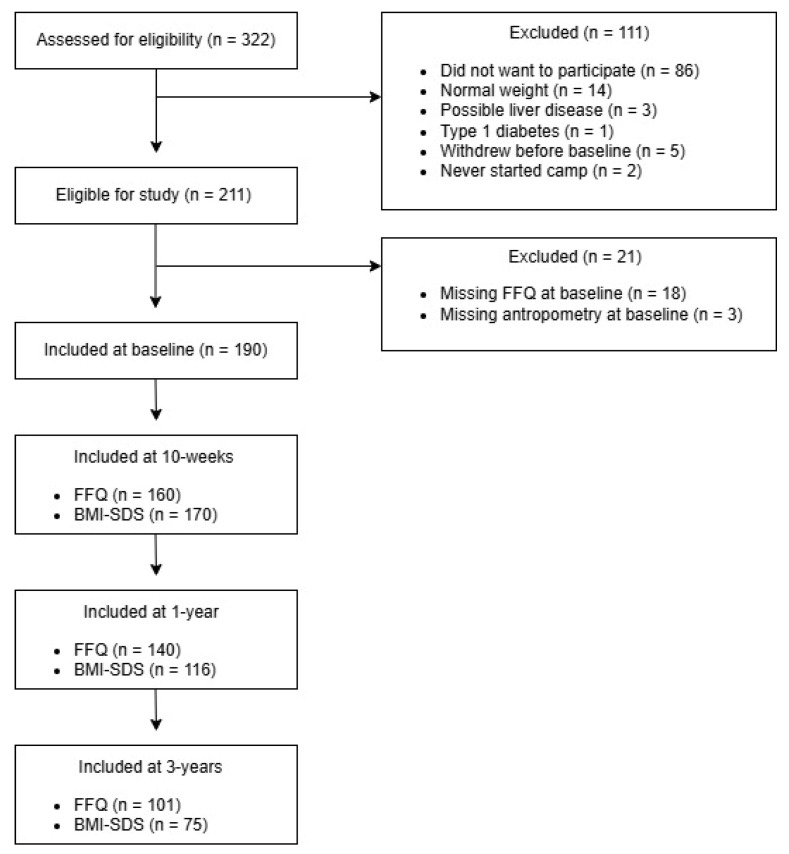
Flowchart of study participants.

In total, 322 children were invited to participate and initially assessed for eligibility. Of these, 93 children declined to participate, withdrew or never started camp. Additionally, 14 children were excluded due to having normal weight at baseline and four due to chronic illness requiring a special diet, resulting in 211 children being eligible. Additionally, 21 children were excluded due to missing FFQ or anthropometric data at baseline (Figure 1). In total, 190 children were included at baseline with a mean age of 12.3 ± 1.4 years, divided into 108 girls and 82 boys. At baseline, the children had a BMI-SDS of 2.62 ± 0.7 SD, a body fat percentage of 41.27 ± 6.6%, and a skeletal muscle mass of 23.23 ± 5.2 kg (Table 2). Beverage intake was not associated with anthropometric measures at baseline.

### 3.1. Changes in Beverage Intake Following Lifestyle Intervention

After the 10-week camp, a lower proportion of children reported a high intake of SSBs (36% vs. 11%), NCBs (64% vs. 37%), and chocolate milk (33% vs. 12%), and a greater proportion reported a high intake of water (54% vs. 74%) compared to baseline. Comparing intake levels from end of camp to the one- and three-year follow-up, the changes attenuated reaching baseline levels for water, SSBs, and NCBs at the three-year follow-up. At the one-year follow-up compared to end of camp, children had 2.81 (95% CI 1.57; 5.04) higher odds of reporting high intake of NCBs, 3.20 (95% CI 1.52; 6.72) higher odds of reporting high intake of SSBs, and 0.46 (95% CI 0.26; 0.82) lower odds of reporting high intake of water relative to a low intake. At the three-year follow-up compared to end of camp, children were more likely to report a high intake of SSBs (OR: 8.65 (95% CI 3.87; 19.36)), NCBs (OR: 6.60 (95% CI 3.28; 13.27)), and chocolate milk (OR: 3.19 (95% CI 1.43;7.12)). Within the same period, children were less like to report a high intake of water (OR: 0.32 (95% CI 0.17; 0.61)) and milk (OR: 0.26 (95% CI 0.13; 0.50)) (Table 3).

In total, few children (13%) moved from the high to the low SSB intake category between baseline and the three-year follow-up, whereas 17 children (19%) shifted from low to high SSB intake during the same period. Similarly, 14% moved from the high to the low NCB intake category between baseline and the three-year follow-up, and 20% shifted from low to high NCB. From end of camp to the three-year follow-up, the majority of children did not change SSB (65%) or NCB (60%) intake category.

### 3.2. Associations Between Changes in Beverage Intake and Anthropometry Across Time

No associations were found between changes in SSB and milk intake and changes in anthropometry measures across time. From baseline to end of camp, an increase in water intake was negatively associated with BMI-SDS (r_s_ = −0.21, *p* < 0.05, Table 4) and skeletal muscle mass (r_s_ = −0.20, *p* < 0.05, Table 4). Additionally, an increase in water intake from end of camp to the three-year follow-up was negatively associated with BMI-SDS (r_s_ = −0.24, *p* < 0.05, Table 4) and positively associated with skeletal muscle mass (r_s_ = 0.25, *p* < 0.05, Table 4). For NCB, an increased intake from end of camp to the one-year follow-up was positively associated with skeletal muscle mass (r_s_ = 0.31, *p* < 0.01, Table 4). For chocolate milk, an increased intake from end of camp to the one-year follow-up was negatively associated with body fat % (r_s_ = −0.19, *p* < 0.05, Table 4), whereas an increased intake from end of camp to the three-year follow-up was negatively associated with BMI-SDS (r_s_ = −0.26, *p* < 0.05, Table 4).

## 4. Discussion

The present study showed that children with overweight and obesity who participated in a 10-week lifestyle camp intervention reduced their intake of SSBs, NCBs, and chocolate milk, while increasing their intake of water during camp, although the favorable changes attenuated over time, reaching baseline levels for water, SSBs, and NCBs at the three-year follow-up. At the three-year follow-up, children had eightfold, sixfold, and threefold higher odds of reporting high versus low intake of SSBs, NCBs, and chocolate milk, respectively, compared with immediately after the intervention. Moreover, increases in intake of water and chocolate milk were associated with reductions in BMI-SDS, whereas increases in intake of water and NCBs following camp were positively correlated with skeletal muscle mass.

As the camps follow national dietary recommendations and furthermore, participants were advised to consume only one beverage containing sugar per week, it was expected that children would report a reduction in SSBs, NCBs, and chocolate milk during this short-term intervention. In accordance with the present findings, existing evidence suggests that the effects of lifestyle interventions on SSB intake tend to diminish over time [23,24]. Similarly, meta-analyses synthesizing evidence on the overall effect of behavioral interventions on child and adolescent health, including weight development, concluded that maintaining positive effects after the intervention remains challenging in the long term [29,30].

To our knowledge, only one previous RCT has found that children continued to reduce their SSB intake beyond the intervention period [22]. It may be speculated whether the cluster-based design used in that study, where entire classes participated, contributed to this sustained effect, as the shared classroom context may have promoted a collective goal of reducing SSB intake rather than changes driven by individuals.

The inverse association between water intake and BMI-SDS, as observed in the present study, are in agreement with most longitudinal studies suggesting that a higher water consumption may reduce the risk of excess weight gain in childhood. However, the evidence remains limited, and cross-sectional studies report more divergent results [31]. The contradictory findings may be explained by differences in study design and the characteristics of the populations assessed. In the present study, only children with overweight and obesity were included, and the lifestyle camp intervention, including dietary changes, led to clinically significant weight loss for all children. Thus, the weak negative association between water intake and skeletal muscle mass may be explained by the fact that weight loss is often accompanied by a reduction in muscle mass [32].

The present study demonstrated a moderate positive association between NCB intake and skeletal muscle mass, which may reflect adherence to the dietary intervention at camp, with participants replacing SSBs with NCBs, interpreted as a healthier alternative. In accordance with this, previous studies indicate that replacing SSBs with NCBs may provide beneficial health effects in both children and adults [17,33].

We identified only one previous study that explored the effect of chocolate milk specifically, and similar to our findings, this study showed that a higher intake of chocolate milk was associated with lower odds of overweight/obesity in 10- to 12-year-olds. Moreover, this study found that children consuming only white milk were 33.1% less likely to have overweight/obesity in comparison to children who were not consuming milk [21]. In the present study, no associations were found between changes in dairy milk intake and anthropometric measures. However, a meta-analysis of RCTs in children suggests that dairy intake in general is associated with favorable changes in body composition [34].

In general, overall diet quality seems to decline during adolescence [35]. Thus, the diminishing effect observed in the present study may also be due to participants’ progression into late adolescence. Moreover, multiple factors beyond beverage intake influence weight development from childhood through adolescence, and more research is needed to explore the causal association between beverage intake and anthropometry.

### Strengths and Limitations

A key strength of this study is the long-term follow-up period, providing both anthropometric measurements and information on beverage intake up to three years after a lifestyle intervention in children. Furthermore, this is one of the first studies to examine chocolate milk as an independent category. However, the results should be interpreted in light of several limitations. The FFQ was used to assess beverage intake, and children may respond inaccurately or provide more socially desirable answers, increasing the risk of biased results. Additionally, the Danish version of this FFQ has not been validated in a population of children with overweight and obesity; however, it has been pilot-tested in a smaller sample of age-matched children to ensure comprehension. Beverage intake was assessed as frequencies, and categorized into ‘low’ and ‘high’ intake, reflecting national dietary recommendations, which limits the ability to capture exact consumption. Although the categorization helped reduce potential sparse data bias given the limited sample size, it limits the level of detail in the analysis. A larger and more generalizable sample size might have allowed a more nuanced examination of intake levels without relying on predefined categories. Moreover, the COPE study was not powered to assess beverage intake specifically, and this, together with the relatively small sample size, may have increased the risk of missing statistically significant associations. Finally, as this is a secondary analysis of a non-randomized controlled study without a control group, conclusions are limited to associations and it is not possible to determine whether the changes observed reflect natural variations in beverage intake.

## 5. Conclusions

Children with overweight or obesity undergoing a short-term lifestyle intervention achieved health-promoting changes in beverage intake, including a reduction in SSBs, NCBs, and chocolate milk; however, the effects attenuated in the long term, reaching baseline levels for water, SSBs, and NCBs at the three-year follow-up, highlighting the need for continuous support. Moreover, increases in water and chocolate milk intake following the intervention were associated with a more favorable development in BMI-SDS; however, more high-quality studies including larger populations of children and adolescents are needed to determine the effect of beverage intake on weight development.

## Figures and Tables

**Table 1:** Categorization of beverage intake.

	Low Intake	High Intake
Water	≤3 times per day	≥4 times per day
SSB	≤1 time per week	≥2 times per week
NCB	≤1 time per week	≥2 times per week
Milk	≤6 times per week	≥1 time per day
Chocolate milk	Never/less than 1 time per week	≥1 time per week

**Table 2:** Baseline characteristics of children included in the COPE study (n = 190).

Sex:	n (%)
Boys/girls	82 (43.2)/108 (56.8)
	**Mean** **±** **SD**
Age	12.3 ± 1.4
Weight	91.1 ± 21
Height	1.70 ± 0.08
BMI-SDS	2.62 ± 0.68
Body fat % (n = 181)	41.27 ± 6.57
Skeletal muscle mass (n = 181)	23.23 ± 5.19
**Socioeconomic Class (Household Income)**	**n (%)**
<USD 31,100 per year	23 (12.1%)
USD 31,100–USD 77,800 per year	83 (43.7%)
USD 77,800–USD 116,700 per year	60 (31.6%)
>USD 116,700 per year	24 (12.6%)
**Distribution by Beverage Intake Categories:**	**n (%)**
**Water**	
Low intake	86 (45.74%)
High intake	102 (54.26%)
**Sugar-Sweetened Beverages**	
Low intake	122 (64.21%)
High intake	68 (35.79%)
**Non-Caloric Beverages**	
Low intake	67 (35.64%)
High intake	121 (64.36%)
**Milk**	
Low intake	87 (45.79%)
High intake	103 (54.21%)
**Chocolate Milk**	
Low intake	127 (67.2%)
High intake	62 (32.8%)

**Table 3:** Longitudinal changes in beverage intake.

	Baseline	10 Weeks	1 Year	3 Years	Baseline (Ref) to 10 Weeks	10-Weeks (Ref) to 1 Year	10-Weeks (Ref) to 3 Years
	n (%)				Odds Ratio (95% CI), *p*-Value		
Water	n = 188	n = 156	n = 137	n = 99			
Low	86 (46%)	41 (26%)	53 (39%)	44 (44%)	Ref.	Ref.	Ref.
High	102 (54%)	115 (74%)	84 (61%)	55 (56%)	3.11 (1.81;5.36), *p* = 0.00	0.46 (0.26;0.82), *p* = 0.01	0.32 (0.17;0.61), *p* = 0.00
Sugar-Sweetened Beverages	n = 190	n = 160	n = 137	n = 96			
Low	122 (64%)	143 (89%)	106 (77%)	60 (63%)	Ref.	Ref.	Ref.
High	68 (36%)	17 (11%)	31 (23%)	36 (37%)	0.14 (0.07;0.28), *p* = 0.00	3.20 (1.52;6.72), *p* = 0.002	8.65 (3.87;19.36), *p* = 0.00
Non-Caloric Beverages	n = 188	n = 158	n = 139	n = 99			
Low	67 (36%)	100 (63%)	64 (46%)	32 (32%)	Ref.	Ref.	Ref.
High	121 (64%)	58 (37%)	75 (54%)	67 (68%)	0.19 (0.11;0.34), *p* = 0.00	2.81 (1.57;5.04), *p* = 0.001	6.60 (3.28;13.27), *p* = 0.00
Milk	n = 190	n = 160	n = 137	n = 98			
Low	87 (46%)	65 (41%)	61 (45%)	60 (61%)	Ref.	Ref.	Ref.
High	103 (54%)	95 (59%)	76 (55%)	38 (39%)	1.37 (0.81;2.31), *p* = 0.24	0.72 (0.41;1.28), *p* = 0.26	0.26 (0.13;0.50), *p* = 0.00
Chocolate Milk	n = 189	n = 159	n = 138	n = 98			
Low	127 (67%)	140 (88%)	114 (83%)	73 (74%)	Ref.	Ref.	Ref.
High	62 (33%)	19 (12%)	24 (17%)	25 (26%)	0.18 (0.09;0.36), *p* = 0.00	1.64 (0.76;3.52), *p* = 0.21	3.19 (1.43;7.12), *p* = 0.01

**Table 4:** Association between changes in beverage intake and changes in anthropometric data across time points (* < 0.05 and ** < 0.01).

	Time Points	BMI-SDS	Body Fat %	Skeletal Muscle Mass
		Coeff (rₛ)	Coeff (r_s_)	Coeff (r_s_)
Water	0 to 10 weeks	* −0.21	−0.03	* −0.20
	10 weeks to 1 year	−0.02	−0.01	0.08
	10 weeks to 3 year	* −0.24	−0.23	* 0.25
Sugar-Sweetened Beverages	0 to 10 weeks	0.08	−0.10	0.08
	10 weeks to 1 year	0.10	0.05	0.02
	10 weeks to 3 year	−0.03	0.01	−0.13
Non-Caloric Beverages	0 to 10 weeks	0.12	0.01	0.06
	10 weeks to 1 year	0.14	−0.04	** 0.31
	10 weeks to 3 year	0.03	0.03	−0.13
Milk	0 to 10 weeks	−0.11	−0.03	−0.03
	10 weeks to 1 year	0.02	−0.04	0.02
	10 weeks to 3 year	−0.13	0.05	−0.12
Chocolate Milk	0 to 10 weeks	−0.01	−0.09	0.06
	10 weeks to 1 year	−0.16	* −0.19	0.02
	10 weeks to 3 year	* −0.26	−0.09	−0.15

## Data Availability

Data described in the manuscript, code book, and analytic code will be made available from the corresponding author (DDP) upon reasonable request.

## Abbreviations

The following abbreviations are used in this manuscript:

WHO	World Health Organization
RCT	Randomized controlled trial
SSB	Sugar-sweetened beverage
BMI	Body Mass Index
NCB	Non-caloric beverages
BMI-SDS	BMI standard deviation score
FFQ	Food Frequency Questionnaire
